# Differential online and offline effects of theta-tACS on memory encoding and retrieval

**DOI:** 10.3758/s13415-024-01204-w

**Published:** 2024-07-31

**Authors:** Sven Paßmann, Sandrine Baselgia, Florian H. Kasten, Christoph S. Herrmann, Björn Rasch

**Affiliations:** 1 Cognitive Biopsychology and Methods, Department of Psychology, Université Fribourg, Rue P.-A.-de-Faucigny 2, 1700 Fribourg, Switzerland; 2 Present Address: Department of Neurology, University Medicine Greifswald, Greifswald, Germany; 3 Centre de Recherche Cerveau & Cognition, CNRS, Toulouse, France; 4 Université Toulouse III Paul Sabatier, Toulouse, France; 5 Experimental Psychology Lab, Department of Psychology, Carl Von Ossietzky Universität, Oldenburg, Germany

**Keywords:** Declarative memory, Theta, Encoding, Retrieval, Transcranial alternating current stimulation, Memory formation, PFC-hippocampus axis

## Abstract

Theta oscillations support memory formation, but their exact contribution to the communication between prefrontal cortex (PFC) and the hippocampus is unknown. We tested the functional relevance of theta oscillations as a communication link between both areas for memory formation using transcranial alternating current stimulation (tACS). Healthy, young participants learned two lists of Dutch-German word pairs and retrieved them immediately and with a 30-min delay. In the *encoding group* (N = 30), tACS was applied during the encoding of list 1. List 2 was used to test stimulation aftereffects. In the *retrieval group* (N = 23), we stimulated during the delayed recall. In both groups, we applied tACS bilaterally at prefrontal and tempo-parietal sites, using either individualized theta frequency or 15 Hz (as control), according to a within-subject design. Stimulation with theta-tACS did not alter overall learning performance. An exploratory analysis revealed that immediate recall improved when word-pairs were learned after theta-tACS (list 2). Applying theta-tACS during retrieval had detrimental effects on memory. No changes in the power of the respective frequency bands were observed. Our results do not support the notion that impacting the communication between PFC and the hippocampus during a task by bilateral tACS improves memory. However, we do find evidence that direct stimulation had a trend for negatively interfering effects during immediate and delayed recall. Hints for beneficial effects on memory only occurred with aftereffects of the stimulation. Future studies need to further examine the effects during and after stimulation on memory formation.

**Supplementary Information:**

The online version contains supplementary material available at 10.3758/s13415-024-01204-w.

## Introduction

The ability to form stable traces of declarative memory is essential to interact successfully with the environment or to form our personality (Squire & Kandel, 2003). The process starts when newly acquired information is processed and stored in corresponding brain regions and in the hippocampus simultaneously (Benchenane et al., 2011). Later, the hippocampus plays an important role for the successful retrieval of stored information even at much later timepoints (Staresina & Wimber, 2019). Notably, second language learners need a functionally unimpaired hippocampus, not only to learn the association between words of a foreign language and their native language, but also to be able to successfully retrieve this association later on (Krishnan et al., 2016).

For such association to be possible, a structural, bidirectional connection between the prefrontal cortex (PFC) and the hippocampus is required (Eichenbaum, 2017), which would need to be established through a suitable medium. Indeed, numerous studies suggest that theta-oscillations (4–7 Hz) may serve as such a medium (Herweg et al., 2020). Theta generators have been found in PFC as well as the hippocampus (Mantini et al., 2007; Vidaurre et al., 2018). Initial evidence for a link between successful encoding of information and increased theta band activity in the PFC was found very early in the field (Klimesch et al., 1996; for review see Hanslmayr & Staudigl, 2014). However, over time, a much more complex picture emerged (Lisman & Jensen, 2013). Nowadays, hippocampal theta waves are assumed to coordinate gamma-encoded information at the temporal level, linking distant cortical areas (Solomon et al., 2017). The importance of this link was demonstrated in several studies (Backus et al., 2016; Burke et al., 2013; Solomon et al., 2017, 2019), showing that the degree of coherence between PFC and the hippocampus influences memory performance positively.

Sirota & colleagues (2008) developed a transmitter–receiver model to explain this process in detail. In this model, theta band activity propagates from the information receiver (hippocampus) to the source structure (PFC) during encoding to coordinate the timing of information transfer by biasing the activity in the source structure. This way, theta oscillations create time windows in which information can be received most effectively (Battaglia et al., 2011), i.e., when the recipient structure is most plastic (Huerta & Lisman, 1996; Hyman et al., 2003). The model further suggests that a precise selection of information is possible through this PFC-hippocampus axis (Benchenane et al., 2011).

Similar to encoding, there is a positive correlation between higher theta power and successful retrieval of episodic (Klimesch et al., 2001a, 2001b; Klimesch et al., 2001a, 2001b) and semantic information (Klimesch et al., 1997). Likewise, it relies on coherence between the PFC and hippocampus (Solomon et al., 2017, 2019), suggesting a mode of information transfer similar to encoding (Hebscher et al., 2019). This seems plausible, because the same brain regions are active during retrieval and encoding (Danker & Anderson, 2010). Moreover, theta activity during retrieval is considered as a clock for cortical reinstatements of distinct memories from the hippocampus to the PFC (Staresina & Wimber, 2019).

Despite all these correlational findings, the question of an actual functional relevance of theta activity in these processes remains open. Transcranial alternating current stimulation (tACS) is an appropriate tool to assess such functional roles because of its potential to alter oscillatory activity of large-scale brain networks and their associated cognitive functions (Klink et al., 2020a, 2020b). tACS works via the application of a weak sinusoidal current noninvasively applied to the scalp. The sinusoidal current modulates intrinsic oscillatory activity by means of neural entrainment (Antal & Herrmann, 2016). Indeed, evidence from animal models suggest that tACS can bias spike timing in a frequency-dependent manner (Fröhlich & McCormick, 2010; Krause et al., 2019).

Notably, with respect to declarative memory, tACS was used in several studies addressing (semantic) associative memory (Alekseichuk et al., 2019b; Antonenko et al., 2016; Klink et al., 2020b; Lang et al., 2019; Marko et al., 2019; Meng et al., 2021). Even though they all applied tACS in the range of the theta frequency band, they followed different stimulation approaches that had a distinct effect on the tACS setting. Antonenko et al. (2016) and Meng et al. (2021) targeted left tempo-parietal brain regions (involved in language learning) showing a benefit in memory, but in different ageing groups (Antonenko et al.: in older participants only; Meng et al.: in young participants). Similar to Antonenko & colleagues, Klink et al. (2020b) targeted the left inferior frontal gyrus (involved in semantic processing) of younger and older participants, but they improved memory successfully in the elderly only. Finally, Alekseichuk et al. (2019b) as well as Lang et al. (2019) targeted the right parietal areas (involved in visual memory) successfully showing a tACS-related benefit in memory of young participants.

However, all the previously mentioned studies did not specifically target the communication of this PFC-hippocampal axis, but only the areas involved in memory formation. Thus, the functional relevance of theta-band activity in relation to this axis for encoding and retrieval of semantic information is still unknown. In particular, it is unclear whether a synchronization of both brain regions is based on this theta frequency band and if an experimental reinforcement of this synchronization supports encoding or retrieval of memories.

To find evidence for the functional relevance of the frequency-based communication between different areas, in-phase stimulation (distant brain areas receive synchronous sinusoidal stimulation) of both areas would be a possible approach (Klink et al., 2020a, 2020b). Similar to declarative memory, frontal as well as parietal areas support working memory (e.g., Pessoa et al., 2002; Todd & Marois, 2004) where oscillatory activity seems to organize the local neuronal ensembles across distant regions during WM processes (Buzsáki, 1996; Sarnthein et al., 1998). Therefore, previous studies of working memory have shown that this form of tACS has a positive influence on behavioral control (Polanía et al., 2012; Violante et al., 2017; Alekseichuk et al., 2017). However, evidence at the neurophysiological level has so far only been provided by means of bold activations in the corresponding areas (Violante et al., 2017), whereas evidence of synchronization between the areas using EEG has only yielded marginal findings (Alekseichuk et al., 2017). Whether a corresponding synchronization of PFC and HPC also is possible in declarative memory has not yet been researched.

In the current study, we applied tACS with individual theta frequency (fixed beta frequency as control) to synchronize the PFC-hippocampus axis either during encoding (*encoding group*) or retrieval (*retrieval group*) of acoustically presented Dutch–German word pairs to improve semantic memory performance. Synchronization was achieved by using two target electrodes on the left hemisphere and two return electrodes on the right hemisphere attached at prefrontal and tempo-parietal sites. Because of this configuration, the two electrodes in each hemisphere oscillate in synchrony (in-phase) to achieve coherence of the two distant brain areas. Since synchrony in the PFC-hippocampus axis is assumed to be beneficial for both encoding and retrieval processes, we hypothesized a benefit in memory performance in favor of theta-based tACS compared with beta-tACS in both the encoding and retrieval group (see preregistration for encoding and retrieval study). Given that electrical stimulation is able to change brain activity, we also assumed that theta-tACS increases power in the respective frequency band from before to after stimulation.

## Methods

### Participants

Healthy, young, right-handed subjects (18–35 years) with German mother tongue (or at least C2 level of proficiency) and without Dutch language skills were recruited via advertisement at the University of Fribourg or in local public places. All completed questionnaires for major exclusion criteria (BDI, Kühner et al., 2007, exclusion if score > 14 points; contraindications for transcranial electrical stimulation; severe untreated medical, neurological and psychiatric diseases; intake of drugs and medication that affect the central nervous system; current pregnancy; nonnative German speakers; shift working in the past 6 weeks; known sleep disturbances). Corresponding surveys showed that none of our participants had taken medication that affects the central nervous system. In the context of this, three subjects in the encoding group and nine subjects in the retrieval group reported smoking cannabis occasionally with the last consumption taking place at least 1 week before inclusion in the study (N = 2, everyone else at least half a year to 1 year). We asked the subjects to abstain from consumption for the duration of the study. During the study, cannabis consumption was checked by enquiry, which was negative in all cases, and thus exclusion was not necessary.

A total of 65 eligible subjects entered the study. Six subjects from each group were excluded because of severe headaches (1 participant from *encoding group* only) and/or technical problems. The final sample of the *encoding group* consisted of 30 subjects (mean age ± SD 22.3 ± 3.6, 24 females) and 23 subjects for the retrieval group (mean age ± SD: 22.2 ± 3.2, 17 females; Table 1), which all completed two experimental sessions in the laboratory of the University of Fribourg. All participants gave written, informed consent before the study and received a small reimbursement. The study was conducted in accordance with the Declaration of Helsinki and was approved by the Swiss Ethics Committee (Swiss Association of Research Ethics Committee). Additionally, the hypotheses, method, and planned analyses of both experimental groups were preregistered with the Open Science Framework before any human observation (*encoding group:* https://osf.io/5fknq*; retrieval group:* https://osf.io/ysw5k).

**Table 1:** General questionnaires, individual theta frequency, and impedance measures

	Encoding group		Retrieval group	
	N = 30 (24 female)		N = 23 (17 female)	
	Mean ± SD		Mean ± SD	
Age (years)	22.3 ± 3.6		22.2 ± 3.2	
D-MEQ	50 ± 9.2		47.9 ± 9.1	
BDI	3.7 ± 3.5		3.7 ± 2.6	
PSQI total	4.3 ± 2.1		4.2 ± 2.1	
	S1	S 2	S1	S2
ITF (in Hz)	5.1 ± 1.1	5.2 ± 1.1	5.2 ± 1	5.3 ± .9
Impedance (in kΩ)	1.7 ± 1.1	1.8 ± 1.2	1.5 ± .9	2.7 ± 4

Significant differences highlighted in bold. SD, standard deviation; D-MEQ, German version of Morning-Eveningness-Quesstionnaire; BDI, Becks Depression Inventory; PSQI, Pittsburgh Sleep Questionnaire Inventory; S1/S2, session 1/session 2; ITF, individually determined theta frequency; Hz, Hertz

### Procedure

**Fig. 1:**
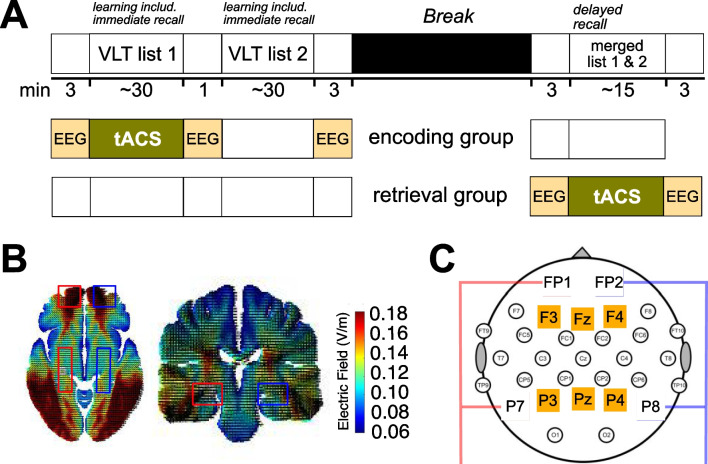
Study design. (**A**) *Time course of the experiment*. In both groups, participants did two sessions with a break of at least one week in-between (one session per tACS-condition). The order of the tACS conditions (theta/beta-tACS) was randomly assigned among participants. They were asked to memorize word pairs from two different lists in each session. During break (30 min), participants completed a finger-sequence tapping task (non-declarative memory) and were offered time to recover. Pale rosé boxes depict timepoints for resting EEG recordings (eyes closed). First resting EEG recording was used to calculate individual theta-frequency. (**B**) *Electric field simulation*. Standard head model using ROAST toolbox for Matlab (roast V2.7.1, (Huang et al., 2019). Rectangles in red and blue display the area of interest (PFC, hippocampus). (**C**) *EEG and stimulation electrode placement*. Electrode placement according to 10–20 international EEG system. Stimulation electrodes were placed at FP1, FP2, P7, and P8. Red boxes depict target electrodes, blue boxes depict return electrodes. TACS was administered with 0° phase difference within hemisphere, and 180° phase difference between hemispheres. Electrodes highlighted in orange were chosen for EEG analysis: F3, Fz, and F4 for frontal region, P3, Pz, and P4 for parietal region. VLT, verbal learning task; tACS, transcranial alternating current stimulation; V/m, Volt per meter; min, minutes

In both the *encoding* and the *retrieval* *group* (within-subject factor), two experimental sessions (each with either with theta- or beta-tACS, between-subject factor) took place either in the morning or in the afternoon, with a break of at least 1 week (mean days ± SD: for *encoding group* 11.3 ± 4.5; for *retrieval group* 11.2 ± 7; Fig. 1A). Because of the Covid pandemic, two participants had a break of approximately 101 and 98 days, respectively, between the two sessions in the *encoding group* and one subject had a break of 38 days between the two sessions in the retrieval group.

Before their first session, participants completed additional questionnaires for handedness (Edinburgh Handedness Inventory (Oldfield, 1971)) as well as subjective sleep habits (Pittsburgh Sleep Quality Index (Buysse et al., 1989); German version of Morningness-Eveningness-Questionnaire (Griefahn et al., 2001); Table 1). Throughout the entire sessions (specifically after tACS administration and/or after learning both lists), experimenters asked participants about their well-being and explained the procedure and the applied techniques to make them comfortable.

Both experimental sessions shared the same protocol. At the beginning, participants completed questionnaires to ensure adherence to previously issued instructions (no caffeine or alcohol intake) about their sleep in the last night (SF-A/R questionnaire (Görtelmeyer, 2011)) and their current mood (“Mehrdimensionaler Befindlichkeitsfragebogen”; MDBF (Steyer et al., 1997)). Afterwards, they were equipped with electrodes for tACS and electroencephalography (EEG). Before starting to learn the first list, a 3-min resting EEG with eyes closed was recorded to determine the individual theta frequency (ITF). Participants were then familiarized with the memory task through a shortened version of the vocabulary learning task. Meanwhile, the ITF was calculated separately for both sessions.

In each session, participants had to learn two of four different lists of Dutch–German word pairs presented in a randomized order over all participants. After another 1-min resting EEG with eyes closed, three runs of learning followed (learning, learning + feedback, and immediate recall). As soon as the first learning round started in the *encoding group*, tACS was initiated in the intended condition (conditions equally distributed, for details see section *Transcranial Alternating Current Stimulation (tACS)*). After finishing all three runs of the first list, a second list of word pairs was presented in the same way as the first list, while EEG was recorded. Before and after learning of the second list, two more resting EEG were recorded with eyes closed (post-STIM1: 1 min before learning; post-STIM2: 3 min after learning) for later analyses. During the time period between immediate and delayed recall, participants completed an adjusted version of a finger-sequence tapping task (Walker et al., 2002) followed by a break and the delayed recall. After finishing the delayed recall participants completed a questionnaire about stimulation-related sensations. At the end of the second session, a postexperiment questionnaire was filled out additionally.

To monitor participants’ tiredness, they completed a 10-point Likert scale in both experimental sessions prior to stimulation (pre-STIM) and after (post-STIM). Participants as well as experimenters were blinded to the stimulation condition throughout the whole testing. The programming of the stimulation device was performed by a laboratory member who had no contact with the participants or was only sometimes involved in session preparation (involved in 14 of 60 sessions in total). In the case of session preparation, the laboratory member did not learn the stimulation conditions until it was time to program the tACS device.

In the *retrieval group*, the procedure and materials used were similar as in the *encoding group* with some exceptions in the protocol. First, tACS in the respective stimulation condition was administered during the delayed recall (11 participants received theta-tACS first). Second, we recorded the 3-min resting EEG episodes for later analyses in the recall session, before and after stimulation. At these timepoints, we also handed out the VAS questionnaire and asked the participants to indicate their level of tiredness. The laboratory staff responsible for programming the device were involved in the preparation eight times (46 sessions in total).

### Behavioral tasks

#### Vocabulary learning

We adopted the vocabulary learning task described in previous studies (Göldi et al., 2019; Schreiner & Rasch, 2015; Schreiner et al., 2018, 2015a, 2015b, Fig. 2) and created four different lists (two lists for each session, 192 word pairs in total) containing 44 Dutch words and their German translations (not included in the analysis). We added two additional word pairs at the beginning and the end to prevent primacy/recency effects. All lists were created equally in terms of Levenshtein scale and letter digits (based on Dutch words only) and were balanced across participants and stimulation conditions. All word pairs were presented acoustically as in Schreiner et al., (2015a, 2015b).

**Fig. 2:**
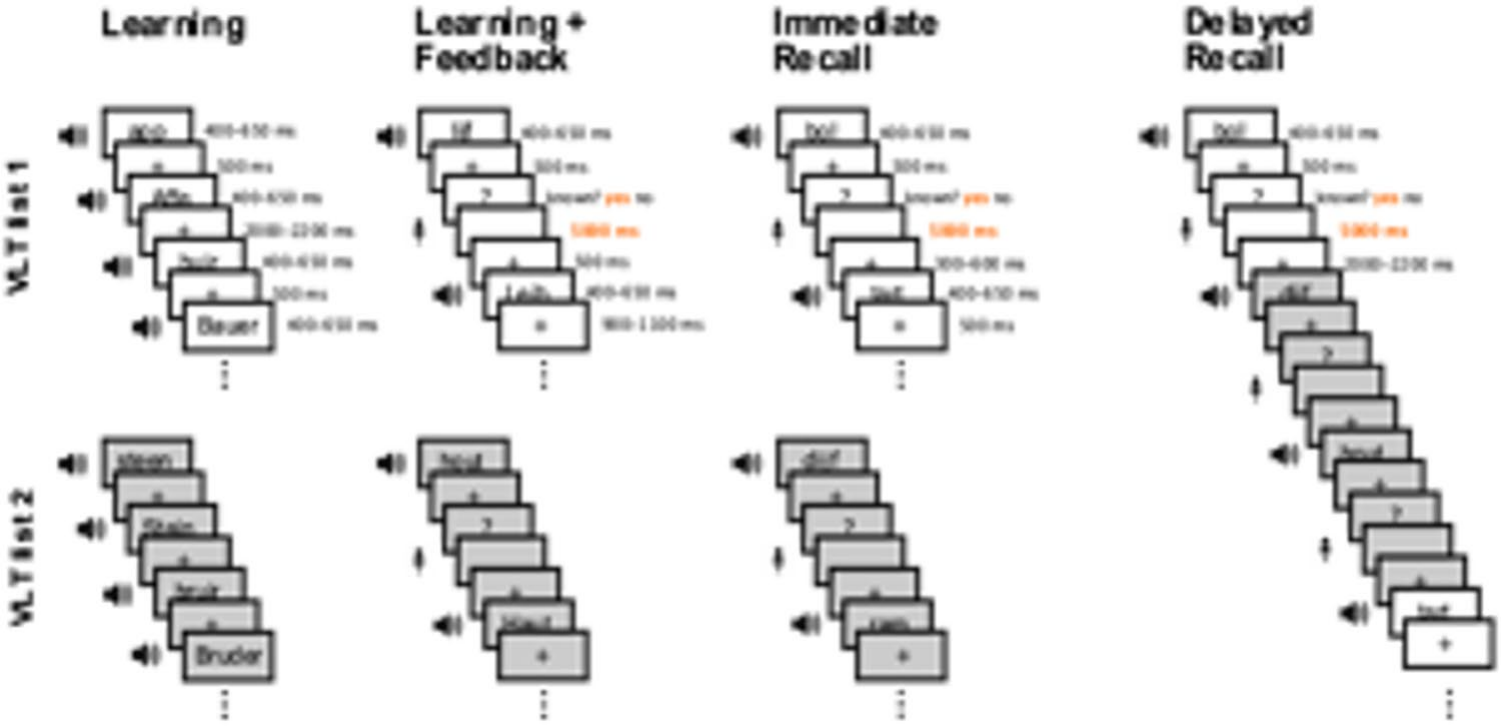
Vocabulary learning task. Subjects learn two different Dutch-German word pair lists in succession with a total of 44 word pairs in each list. The learning of each word pair list has three phases: (1) A learning phase in which the word pairs are presented acoustically and the subjects are asked to memorize the word pairs. (2) A learning + feedback phase, where the Dutch word of a pair is presented acoustically. Participants are asked whether they remember the corresponding German word. If so, the German word should be spoken aloud while the spoken word is recorded. Regardless of the answer, the German word is then presented again acoustically. (3) An immediate recall phase, with the same procedure as in phase 2, but without another acoustic presentation of the corresponding German word at the end. After a break of 30 min, the delayed recall takes place, whereby both lists are mixed in accordance with the procedure of the third learning phase

The learning of the word pair lists consisted of three runs: learning, learning + feedback, and immediate recall. During the initial encoding, the participants listened to both words of each pair following the instruction to memorize the presented word pairs as well as possible. In the second round, they first heard the Dutch word, followed by the question whether they remembered the corresponding German word, which, in case of a positive answer, were expected to pronounce the word aloud. Independent of their answer, the German word was presented acoustically afterwards as feedback. During immediate recall, the same sequence as in the second run took place without giving feedback for the correct answer.

After a 30-min break, we cued the participants with the Dutch words to recall as many of the corresponding German translations as possible from both lists presented acoustically in a randomized order. Here, as well as during immediate recall, we recorded the pronounced answers digitally. The stimuli were presented on a 24-inch screen using the application E-Prime (Version 2.0.10).

#### Finger-sequence tapping task

Between the last immediate and the delayed recall (30 min later), we applied a distractor task in order to keep participants alert during the break. The task consisted of the to-be-learned-part of a finger-sequence tapping task only (procedural memory (Walker et al., 2002)). In twelve 30-s runs with 30-s breaks in-between, participants were asked to tap a given sequence of numbers (4–2-1–3-4 or 2–4-1–3-2) as fast and as accurately as possible on a keyboard with their nondominant (left) hand. The data have not been recorded. The rest of the 30-min break was used to offer time for recovery.

### Transcranial alternating current stimulation

To target PFC and the hippocampus and to synchronize both areas in each hemisphere, we attached four rectangular rubber-electrodes (5 × 5 cm) over left and right PFC (FP1/FP2) as well as both parietal regions (P7/P8) according to the 10–20 international EEG system with conductive paste (Ten20, D.O. Weaver, Aurora, CO; Fig. 1C). This set up was chosen to ensure the greatest possible current in the target areas. Therefore, we simulated 12 different electrode configurations, which included both 3-electrode (two target, one return) as well as four-electrode approaches (two target, two return). For the simulations on a standard head model using the ROAST toolbox for Matlab (roastV2.7.1; Huang et al., 2019; Fig. 1B), frontal (FP1, FP2, F4), central (Afz), parietal (P7, P8), as well as tempo (TP7, TP8)- and centroparietal electrode positions (CP5, CP6) have been included to deliver the electric current as close as possible to the designated brain areas (see supplemental Table S1 for more details).

In detail, the stimulation electrodes on the left hemisphere (FP1 & P7) served as the target electrodes and the respective electrodes on the right hemisphere (FP2 & P8) served as the return electrode in order to synchronize PFC and hippocampus. While the current oscillated with 0° phase difference within each hemisphere (in-phase, Klink et al., 2020a, 2020b), there was a 180° phase difference between hemispheres (anti-phase). There are no other differences between the electrodes. To split up the current delivered by one battery-driven device (DC-Stimulator, neuroConn GmbH Ilmenau, Germany), we used a splitterbox (Medizin Technik Berger, Oldesloe/Germany), which splits each cable into two further channels (two each for both target and return electrode). The peak-to-peak intensity amounted to 2 mA. The impedance in both groups was kept below 5 kΩ (see Table 1 for impedance values).

While participants did the task training, we calculated the stimulation frequency in the theta band for each participant individually, using the first of the 3-min resting-EEG recordings. Using an approach described first by Klimesch & colleagues (1996), a Matlab-based script (Corcoran et al., 2018) computed the individual’s theta frequency (ITF) by subtracting 5 Hz from the determined individual’s alpha peak (Table 1). This approach is based on findings showing that the theta frequency band varies as a function of the alpha frequency which therefore can serve as a reference point for the determination of, among other things, the theta range (Klimesch, 1999). Fixed values for stimulation were used in cases no alpha peak could be detected (5 Hz, instead of ITF), which was the case for three participants in the *encoding group* in the session theta-tACS was applied, whereas in the *retrieval group* only one participant showed no clear alpha peak in the respective session. For the control condition, a fixed frequency of 15 Hz was used.

During the experiments, tACS was applied for 23 min during the presentation of the first list in the *encoding group* and for about 15 min during the delayed recall in the *retrieval group*. Both time periods were chosen to cover either the average time needed to learn a list from initial learning to immediate recall (*encoding group*) or the average time needed to recall all items (*retrieval group*). Hereby, the average time for learning one of the two lists or the delayed recall of both lists is calculated from the times in which the items are presented and the response behavior of the participants. While the presentation of the learning round is fixed in time (based on the length of the words presented, interim times, etc., see Schreiner et al., 2015a, 2015b), because no response behaviors of the subjects is necessary here, the duration of the learning + feedback and the immediate recall rounds depends on how many items have been categorized as known. Either all cues are answered, both in the learning + feedback and the immediate recall round or none of the cues are recognized as known. Similarly, the average duration to complete the delayed recall is calculated.

It should be mentioned that there is an ongoing debate about whether the consolidation of information (reflected in the learning + feedback and immediate retrieval rounds in classical learning paradigms) is part of the encoding of information or whether this should be considered as a separate process (Roediger III & Uner, 2022). Because we are interested in the whole process of learning new information (encoding and consolidation), which requires theta-based communication between PFC and hippocampus all the time, we decided to apply tACS in all parts of the process.

In both groups, the sinusoidal current was administered in the respective stimulation condition with either an individually determined theta or a fixed beta frequency as control (see section *EEG recordings and preprocessing*) and with 10-s ramp-in/-out for theta-tACS and 100 cycles for control (owing to technical reasons). In cases of a premature end of the task, the stimulation was stopped manually.

### EEG recordings and preprocessing

Brain activity was recorded (ground: FCz, impedance < 5 kΩ, reference: AFz, bandpass: 0.3–70 Hz, 500 Hz digitization rate) during periods of resting with eyes closed (before and after tACS in both groups, as well as after the learning of the second word pair list in the *encoding group* only) with BrainVisionRecorder Version 1.21 (Brain Products GmbH, Munich, Germany) and stored for later offline analyses. For two participants, the EEG of the third time point was not recorded due to mistakes by the testers. Sintered Ag/AgCl ring electrodes were mounted into an EASY cap at 27 scalp sites according to the extended 10–20 international EEG system using a BrainAmp amplifier system (Brain Products GmbH, Munich, Germany). Additionally, electrodes for EOG (one electrode below the right eye) and ECG were attached.

The preprocessing of resting EEG data was implemented with FieldTrip (Oostenveld et al., 2011), a Matlab-based toolbox (Version R2018b, (MATLAB, 2018*. 9.5.0.944444*, 2018)). The 1 min EEG recordings before and after the encoding of each of two lists (*encoding group*) as well as before and after the delayed recall (*retrieval group*) were visually inspected for artifacts. In order to keep all resting EEG segments comparable, we selected 1 min from the longer segments in which on average the least artifacts were observed. Afterwards, all 1 min epochs were filtered (low cut: 0.5 Hz; high-cut: 35 Hz) and re-referenced to averaged mastoids. After segmentation in equally sized segments (4 s), a Hanning window (100%) was applied on up to nine nonoverlapping artifact-free segments. FFT spectra (mean power, ${\mu }^{2}$) were computed and averaged for the main frequency bands of interests: theta (ITF ± 1 Hz, based on calculation in the theta-tACS session) and beta (15 Hz ± 2 Hz), as well as delta (1–4 Hz) and alpha band (8–12 Hz). Since stimulation electrodes were attached to FP1/FP2 and P7/P8, respectively, we defined the regions of interests (ROI) for power analyses as the electrodes close to them: F3, Fz, F4 for frontal, P3, Pz, P4 for parietal.

### Statistical analyses

All statistical analyses were performed with R (version 4.0; Team R. Core., 2019). The one-sided level of significance (directed hypotheses) was set to α = 0.05 in all analyses and the reported effect size is *η*^*2*^. Significant *p*-values related to tACS-related effects were corrected by using the Holm-Bonferroni stepwise approach (Holm, 1979), with only corrected *p*-values (p_c_) < 0.05 considered significant.

In addition, we conducted Bayesian statistical analyses to quantify the plausibility of alternative H_1_ compared with the null hypothesis H_0_, with the Bayes factor (BF) as a result variable representing a measure of comparability between the null model and all other models. Effects are reported as the Bayes factor for the inclusion (BF_incl_) of a particular effect, which is the ratio between the likelihood of the data given the model compared to the model without that specific effect (see Keysers et al. (2020) for detailed description of BF_incl_) across matched models (see Supplemental Tables S3-8 for detailed information). Here, the level of evidence (see also Stefan et al. (2019)) is classified inconclusive/anecdotal for BF between 0.33 and 3, moderate for BF < 0.33 or > 3, strong for BF < 0.01 or > 10, very strong for BF < 0.03 or > 30, and extreme for BF < 0.001 or > 100. For the Bayesian repeated measures ANOVAs, we used the JAMOVI package (version 2.5.3) with its default priors, including the jsq Bayesian Methods package (version 1.2.0).

#### Vocabulary learning task

Based on the pre-registration, a 2-factorial repeated-measures analysis of variances with the within-subject factors STIM (theta-/beta-tACS) and TIME (immediate/delayed recall) was conducted on the total number of correct answers (sum of *Hits* in both lists; *Hits*: precise naming, in singular or plural form). Because of a more efficient result representation, we analyzed both groups simultaneously and added the factor GROUP to our analysis.

In the exploratory analyses for *the encoding group*, we extended this approach and separated the *Hits* by lists—stimulation during learning (online) vs. learning after the stimulation (offline)—to clarify whether the effect of stimulation was restricted to the time of stimulation itself or whether it would last beyond the stimulation. Thus, we added the factor LIST (online/offline). For the *retrieval group*, we used the same analytical approach.

#### Spectral power

With respect to oscillatory activity in resting EEG, we analyzed the impact of tACS on log-transformed power values via linear mixed models with R-package lme4 (Version 1.1–29, (Bates et al., 2015)). For each frequency band of interest and ROI (see EEG recording and preprocessing section), the computation was done based on this model:

$${y}_{ij}= {\beta }_{0}+{TIMEx}_{1ij}+ {ORDERx}_{2j}+ {STIMx}_{3ij}+ {STIM* TIMEx}_{4ij}+ {u}_{0j}+ {\varepsilon }_{ij}$$

where *y*_*ij*_ is the specific frequency band value at time point *i* of subject *j,* ${\beta }_{0}$ is the fixed effect intercept, ${x}_{1ij}$ is the value for time point centered (values: − 1, 0, 1) at time point *i* in subject *j*, ${x}_{2j}$ is the value for ORDER of stimulation (1: first session theta-tACS, second session beta-tACS; 0: first session beta-tACS, second session theta-tACS) in subject *j*, ${x}_{3ij}$ is the value for STIM (values for theta-tACS: 1, for beta-tACS: 0) for time point *i* and subject *j*, ${x}_{4j}$ is the value for the interaction for STIM and TIME for subject *j*, ${u}_{0j}$ is the residual or random effect for the intercept for subject *j* (mean 0, variance ${\sigma }_{u0}^{2}$), ${\varepsilon }_{ij}$ is the error term for time point *i* and subject *j* (mean 0, variance ${\sigma }_{\varepsilon }^{2}$).

For the *encoding group*, we analyzed stimulation-induced effects in 3-min resting EEG periods at time points before and after tACS (TIME: pre-STIM, post-STIM1, post-STIM2) serving as level-one units nested in subjects (level-two units). Note, that we added post-STIM1 (1-min resting EEG only) to the pre-registered time points pre-STIM & post-STIM2 in order to test the impact of tACS immediately after stimulation as well. Differences between the two tACS conditions were tested by random intercept models (STIM), while the interaction STIM x TIME assessed whether the slopes of the curves differed between conditions. All models were adjusted for sequence of stimulations (ORDER). Every stimulation effect is reported based on model-based estimations. Effects of TIME & STIM × TIME-interaction- and sequence-values are based on regression coefficients (*β*).

For the *retrieval group*, we used the same model as in the *encoding group*, limited to two levels of the factor TIME (pre-/post-STIM).

#### Questionnaires

For the *encoding* and *retrieval group,* we analyzed the impact of stimulation on the individual level of tiredness before and after stimulation for confounding effects. This was analyzed with a 3-factorial rmANOVA approach with the factors GROUP (*encoding/retrieval group*), STIM (theta-/beta-tACS), and TIME (pre-/post-STIM).

We also checked any possible differences of mood (MDBF) and sleep quality the night before (SF-A/R) between single sessions and groups by a 2-factorial rmANOVA approach with the factors GROUP (*encoding/retrieval group*) and SESSION (session1/session2), as well as between GROUP and STIM (theta-/beta-tACS). Any influences on encoding performances were assessed by Pearson’s correlations if indicated.

## Results

### Effects on general memory performance

According to our main hypothesis, we expected increased memory performance with theta-tACS compared with the control stimulation. For the *encoding group*, increased memory performance should be visible both during immediate and delayed recall. For the *retrieval group*, we expected memory increases particularly in the delayed recall (as no stimulation occurred during immediate recall).

**Fig. 3:**
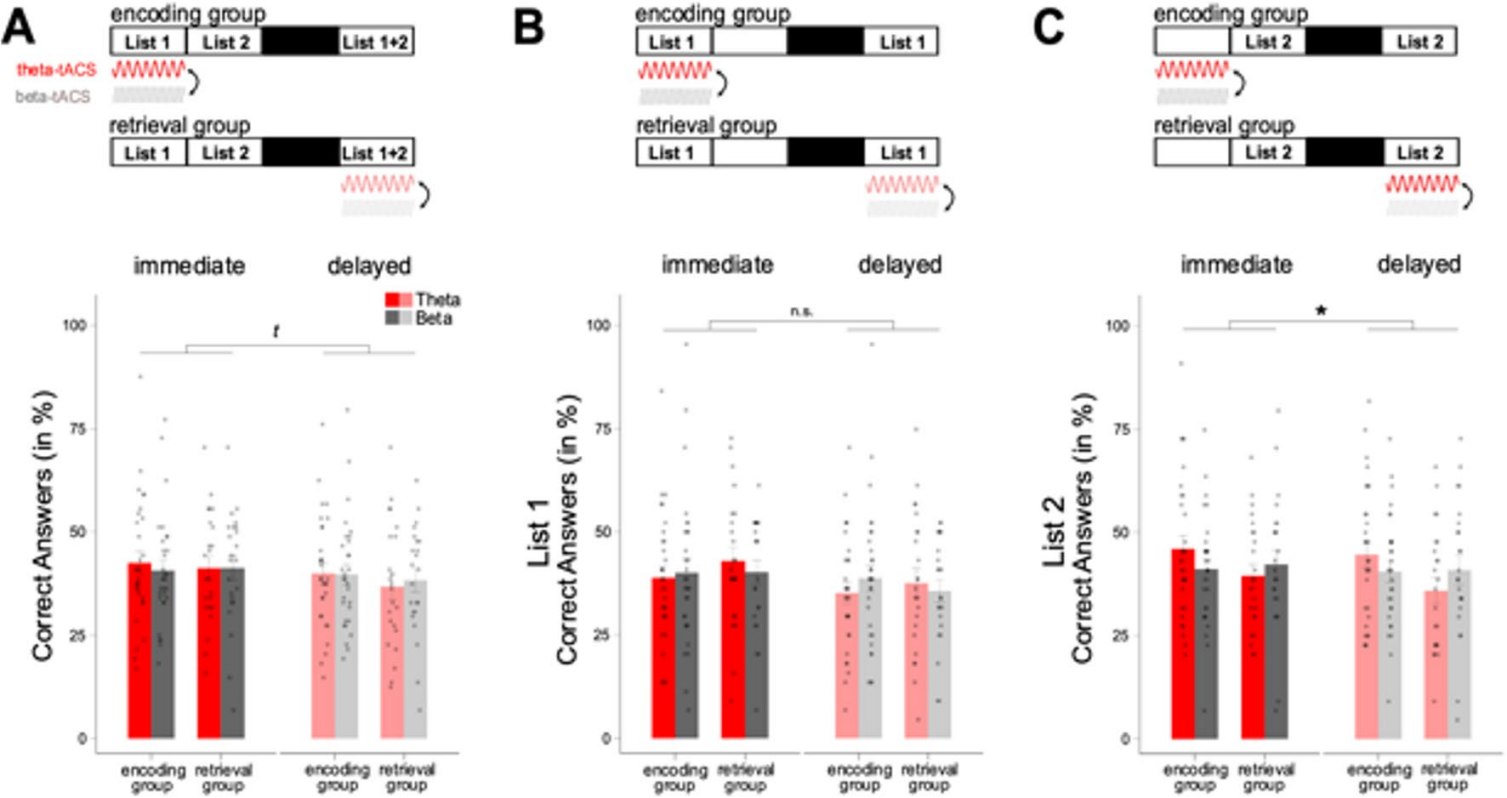
Behavioral results. (**A**) The performance (correct answers in %) in the language learning task of both groups during immediate (vivid colour) and delayed recall (pale colour), separately for tACS-conditions (red: theta-tACS; grey: beta-tACS). A simplified representation of the study design is shown above the results (as well as above **B** and **C**). Boxes containing list numbers represent the respective word pair list(s) whose data are presented in the graphic below (**A**: both lists; **B**: list 1; **C**: list 2). No tACS-related effects were found with respect to memory performance, neither in the *encoding* nor *retrieval group*. However, the change in performance over time was significant, in both groups. (**B**) The memory performance of *list 1*, in both groups, where we found no tACS-related effect, but a general change over time on a significant level. (**C**) The analysis of *list 2* revealed a significant advantage for theta-tACS compared to control stimulation with a clear advantage for *encoding group* compared with *retrieval* group. In addition, we also found significant changes in performance over time. *Encoding group*, N = 30. *Retrieval group*, N = 23. offline, learning after tACS administration

Contrary to our hypothesis, we did not observe evidence for general memory improvements with theta-tACS (Fig. 3A; see Table 2 for descriptive information; see supplemental Table S2 for additional correlational analyses). First, the main effect of stimulation type on general memory performance was not significant (STIM: theta- vs. beta-tACS; *F*_*(1,51)* _= *0.01, p* = *0.926, η*^*2*^ < *0.001, BF*_*incl*_ = *0.145*). Second, the interaction between the factors STIM and TIME (immediate vs. delayed recall) revealed a marginal trend in the opposite direction as predicted (*F*_*(1,51)* _= *3.86, p* = *0.055, η*^*2*^ < *0.001, BF*_*incl*_ = *0.287*). This indicates that forgetting (i.e., change in performance from immediate to delayed recall) was marginally higher in the theta-tACS condition compared with the control stimulation, which applied to both the *encoding* and the *retrieval group*. When analysing the two stimulation conditions separately, the effect of TIME was highly significant in the theta-tACS condition (*F*_*(1,52)* _= *33.78, p* < *0.001, η*^*2*^ = *0.014, BF*_*incl*_ = *13,348.545*) and in the control stimulation (*F*_*(1,52)* _= *8.92, p* = *0.004, η*^*2*^ = *0.005; BF*_*incl*_ = *7.993*), which again applied to both groups. There was no significant interaction, including the factor GROUP (tACS stimulation at encoding vs. retrieval, *F*_*(1,51)* _= *0.46, p* = *0.501, η*^*2*^ = *0.001, BF*_*incl*_ = *0.335;* for remaining BF_incl_, see supplemental Tables S3-5).

**Table 2:** Descriptive information on behavioral performance, separately for group, time, and list

	Encoding group		Retrieval group	
	N = 30 (24 female)		N = 23 (17 female)	
	Mean ± SD		Mean ± SD	
Hits immed theta	42.5 ± 15.1		40.3 ± 14	
Hits delay theta	40 ± 13.8		36 ± 15.3	
Hits immed beta	40.7 ± 13.5		40.2 ± 14.6	
Hits delay beta	39.7 ± 13.5		37.4 ± 13.7	
	List 1	List 2	List 1	List 2
Hits immed theta	38.9 ± 15.7	46.1 ± 17.3	41.8 ± 16.8	38.6 ± 14
Hits delay theta	35.3 ± 14.4	44.7 ± 16.6	36.7 ± 17.5	35.2 ± 15.3
Hits immed beta	40.2 ± 18.8	41.2 ± 13.5	39.4 ± 14.3	41.1 ± 17.4
Hits delay beta	38.9 ± 17.4	40.6 ± 13.8	35 ± 13.2	39.8 ± 17.3

Average behavioral performance in the Dutch-German word pair task. *Top* Performance depicted as mean and standard deviation, separated by group and time for the general analyses. *Bottom* Performance depicted as mean and standard deviation, separately for group, time and list for the exploratory analyses. immed, immediate recall; delay, delayed recall

Table 2 Descriptive information on behavioral performance, separately for group, time, and list.

### Explorative analysis: online vs. offline effects of tACS on memory performance

In addition to our primary analysis, we explored possible differential effects of theta stimulation during or before encoding of word-pairs. In the *encoding group*, one word-pair list was encoded during the tACS stimulation, while another list was encoded after the tACS stimulation (factor LIST: online vs. offline encoding). We contrasted these online and offline conditions against the *retrieval group*, where participants learned both lists in absence of stimulation. Note that the distinction between online and offline encoding is arbitrary in the *retrieval group*, because no tACS stimulation was applied during encoding. Thus, we added the factor LIST to the previous analysis (for descriptive information see Table 2, and for additional correlational analyses see supplemental Table S2).

Interestingly, our results showed a GROUP x STIM x LIST interaction (*F*_*(1,51)* _= *4.85, p* = *0.032, η2* = *0.011; BF*_*incl*_ = *48.304*), which speaks for an influence of theta-tACS on the performance dependent on the timing of the list to be learned relative to tACS administration. In addition, we observed a LIST x TIME interaction (*F*_*(1,51)* _= *7.26, p* = *0.01, η2* = *0.001; BF*_*incl*_ = *0.239*), showing larger decrease in performance from immediate to delayed recall in *list 1* compared with *list 2*. Over both learning and recall timepoints, memory performance was better for *list 2* compared with *list 1* (main effect LIST: *F*_*(1,51)* _= *4.49, p* = *0.039, η2* = *0.007*; *BF*_*incl*_ = *11.300;* all remaining *p* > 0.244).

To examine the GROUP x STIM x LIST interaction further, we analysed each list separately with the factors GROUP, STIM and TIME. Importantly, we observed a significant GROUP x STIM interaction for *list 2* (*F*_*(1,51)* _= *5.04, p* = *0.024, η2* = *0.018; p*_*c*_ = *0.048; BF*_*incl*_ = *110.121;* Fig. 3C), showing a benefit on memory performance only for theta-tACS in the *encoding group* but not in the *retrieval group*. The benefits of theta-tACS were visible during both immediate and delayed recall timepoints in the *encoding group*. Thus, the benefit of offline theta-tACS improved memory performance during encoding of the information, and this benefit was maintained during delayed recall. The results pattern resembles our prior hypothesis for the effects of theta-tACS on general memory performance for the *encoding group*.

In contrast to *list 2* (offline), we observed no selective benefit of theta-tACS on memory performance for *list 1* (online). All interactions and main effects with the factor STIM were nonsignificant (*p* > 0.16; Fig. 3B). In addition, we found a significant decrease in performance over time in both lists (*list 1*: *F*_*(1,51)* _= *34.81, p* < *0.001, η2* = *0.013*; *BF*_*incl*_ = *3.75*; *list 2*: *F*_*(1,51)* _= *11.15, p* = *0.002, η2* = *0.003; BF*_*incl*_ = *0.401;* for remaining BF_incl_, see supplemental Tables S6-8).

### Spectral power

**Fig. 4:**
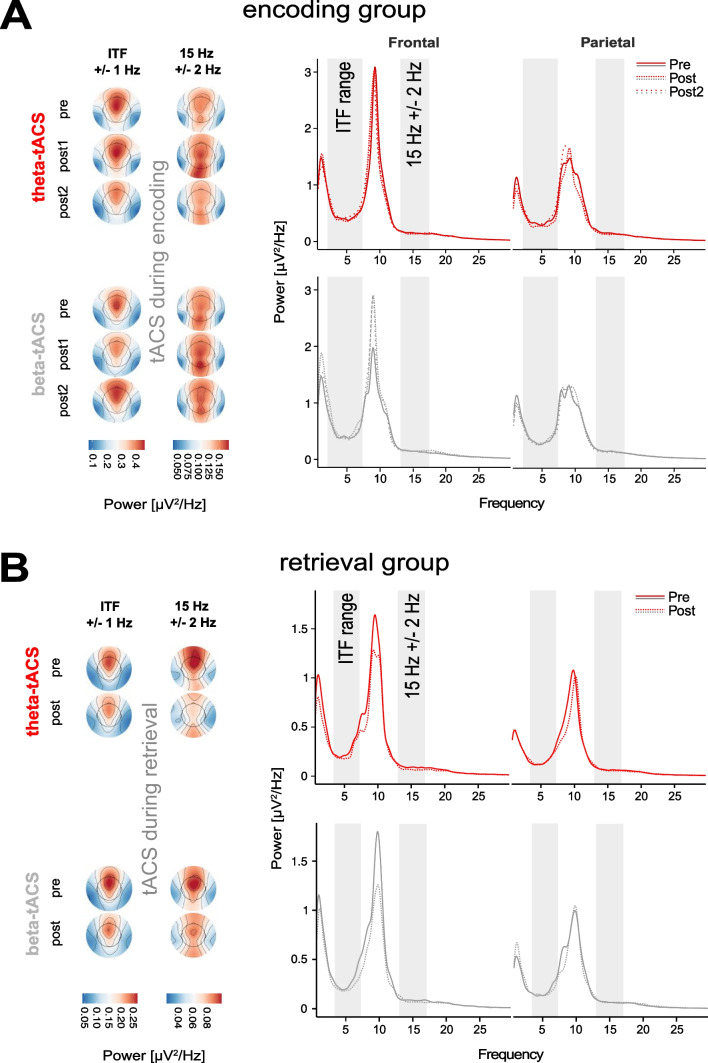
Neurophysiological results. The EEG power during times of resting with eyes closed (**A**, *encoding group*; **B**, *retrieval group*). *Left side*, topographic plots, *Right side*, power spectra. Power averaged across all participants was calculated separately for theta (individually determined theta frequency ± 1 Hz) and beta (15 Hz ± 2 Hz) bands and for the tACS-conditions. For the power spectra, the power was additionally averaged over F3, Fz, F4 for the frontal site and over P3, Pz, P4 for the parietal site. No tACS-related effect at the neurophysiological level in both groups except for the effect over time. Note that for the last time point in the *encoding group*, we only considered data from 28 subjects. *Encoding group*, N = 30. *Retrieval group*, N = 23. ITF, individually determined theta frequency; ITF range, range of individually determined theta frequency in the respective group; Hz, Hertz

We analyzed the power spectra before and after the participants received tACS in the respective main frequency bands and ROI with a Linear Mixed Models approach with the factors STIM (theta-/beta-tACS), TIME (*encoding group:* pre-STIM, post-STIM1, post-STIM2; *retrieval group*: pre-/post-STIM), ORDER (theta-tACS first/beta-tACS first) as well as the interaction of STIM x TIME (Fig. 4).

For both frequency bands of interest namely theta and beta, the *encoding group* showed no tACS-related effects on brain activity before and after immediate recall (*p* > 0.233). For the remaining frequency bands (delta and alpha), we found no impact of STIM or a change over time (*p* > 0.124). However, delta band activity is significantly affected by the order of stimulation with an advantage for theta-tACS first compared to beta-tACS first (factor ORDER: frontal: *β* = *0.17, SE* = *0.08, z* = *2.11, p* = *0.035*; parietal: *β* = *0.22, SE* = *0.09, z* = *2.44, p* = *0.014*; all remaining *p* > 0.06).

For the *retrieval group* during delayed recall, only the decrease in theta-band activity over time at frontal sites is of interest (factor TIME: *β* =  *− 0.12, SE* = *0.04, z* =  *− 2.54, p* = *0.011*). With respect to the remaining frequency bands, delta (factor TIME: *β* =  *− 0.13, SE* = *0.05, z* =  *− 2.75, p* = *0.006*), as well as the alpha band showed a significant reduction of activity over time (factor TIME: *β* =  *− 0.16, SE* = *0.05, z* =  *− 3.16, p* = *0.002*; all remaining *p* > 0.06).

In sum, we could not find a clear influence of tACS on the oscillatory activity in the two frequency bands of interest (theta and beta) at any region of interest, as well as in the remaining frequency bands.

### Questionnaires

In the beginning of each experimental session, participants completed questionnaires related to mood (MDBF) and subjective sleep quality the night before (SF-A/R) analyzed based on a 2 × 2 repeated measures ANOVA with the factors GROUP (*encoding/retrieval group*) and TIME (session 1/session 2, see supplemental Table S9 for pairwise comparison). With respect to MDBF, we only found a significant interaction for rest/unrest (*F*_*(1,51)* _= *5.90, p* = *0.019, η2* = *0.03*), indicating larger feelings of unrest in session 1 (vs. session 2) in the *encoding group* while showing the opposite in the *retrieval group* (all remaining *p* > 0.169). This difference though had no impact on the behavioral performance, neither with respect to the session number (all *p* > 0.110) nor with respect to tACS conditions (all *p* > 0.170). The second analysis based on the tACS condition (GROUP x STIM) revealed significance only in terms of good/bad mood (STIM: *F*_*(1,51)* _= *6.24, p* = *0.016, η2* = *0.03;* STIM x GROUP*: F*_*(1,51)* _= *6.24, p* = *0.016, η2* = *0.03;* all remaining *p* > 0.200; see supplemental Table S10 for pairwise comparisons). In the *encoding group*, correlational analysis revealed that the better the mood, the better the performance in the theta session (r(51) = 0.39, *p* = 0.032). For the *retrieval group*, the analysis showed the opposite, indicating that the worse the mood, the better the performance, which was the case for the second session (r(51) =  − 0.56, *p* = 0.005) and the beta-tACS session (r(51) =  − 0.49, *p* = 0.02, all remaining *p* > 0.079).

Most of the items of the SF-A/R questionnaire revealed no significant effects at all (*p* > 0.097). Some items showed differences in the interaction between groups and sessions that almost reached a level of significance. Participants of the *encoding group* had more difficulties to fall asleep in session 2 (vs. session 1) compared with the *retrieval group* (GROUP x SESSION: *F*_*(1,51)* _= *3.95, p* = *0.052, η2* = *0.03*), whereas in the same group, the sleep quality was better before session 2 (vs. session 1) compared with the *retrieval group* (GROUP x SESSION: *F*_*(1,51)* _= *3.97, p* = *0.052, η2* = *0.03*). The analysis based on stimulation condition (GROUP x STIM) revealed no significance at all (all *p* > 0.114).

With respect to the level of tiredness, participants completed a 10-point Likert scale before and after tACS was applied. In sum, we found no significant influence of tACS on the degree of tiredness that may have had a passive effect on performance (all *p* > 0.171). Please note that in the *encoding group*, seven subjects were removed because they did not complete all respective questionnaires.

### Perception of stimulation

In general, all participants tolerated the stimulation very well. However, more participants reported feelings of tiredness, burning sensations, and to some extent pain under stimulation electrodes with theta-tACS compared with control stimulation. In the remaining aspects (concentrations difficulties, nervousness, pain under stimulation electrodes, phosphenes, tickling and tingling sensations, as well as visual and other unspecified sensations), more participants reported sensations in the control stimulation compared with theta-tACS. No further differences were found. Those subjects who perceived a specific sensation were asked afterwards to indicate the strength of the sensation (see supplemental Table S11). Only in the *encoding group*, subjects reported stronger phosphenes and visual sensations in the control stimulation compared with theta-tACS (phosphenes: *t*_*(29)*_ =  *− 3.41, p* = *0.002*; visual sensations: *Z* = *2.78, p* = *0.006*). No further differences were found.

## Discussion

In this study, we tested whether theta-band activity can enhance memory performance by increasing theta-synchrony between prefrontal cortices and hippocampal structures using tACS. An active stimulation (beta-tACS) served as control condition. tACS was applied during the encoding phase (*encoding group*) and the retrieval phase (*retrieval group*) in different study groups, who learned and retrieved Dutch-German word pairs in two separate sessions (immediate vs. delayed recall).

Contrary to our hypothesis, we found no tACS-dependent effect on general memory performance. We even observed a trend for a higher forgetting (change from immediate to delayed retrieval) in the theta-tACS condition compared with the active control stimulation, speaking against the assumed strengthening of memory stabilization process by electrically increasing theta-synchrony between PFC and hippocampus.

However, in the exploratory analysis, we observed evidence for increased general memory performance in the theta-tACS stimulation when considering only word-pairs that were learned after the stimulation (offline, *list 2*). This effect occurred only in the *encoding group*, as no stimulation was applied during the encoding phase in the *retrieval group*. With the memory performance of the word pairs learned during the stimulation (online, *list 1*), we did not find such result pattern. Thus, aftereffects of theta-tACS stimulation might be beneficial for processes of memory encoding, and these effects are still present during delayed recall. In contrast, direct theta-tACS appears not to have beneficial effects on memory encoding and retrieval.

Our study is the first of its kind in the field of long-term memory that tried to target the PFC- hippocampus axis with in-phase tACS to improve memory performance during encoding and retrieval. We stimulated two brain areas (PFC, hippocampus) per hemisphere with a phase difference of 0° within (in-phase) and 180° between each hemisphere (anti-phase), with the goal to synchronize the respective brain areas per hemisphere. This specific stimulation setup was chosen, because several studies clearly demonstrate that the degree of coherence between PFC and the hippocampus influences memory performance during encoding and retrieval (Backus et al., 2016; Burke et al., 2013; Solomon et al., 2017, 2019). So far, in-phase tACS has been used successfully in studies on working memory. Some studies reported benefits in reaction times in working memory tasks with in-phase stimulation (Hu et al., 2022; Polanía et al., 2012; Violante et al., 2017). In addition, Alekseichuck et al. reported that synchronizing theta oscillations in frontal and parietal areas of each hemisphere benefitted working memory performance (Alekseichuk et al., 2017). Furthermore, Reinhart and Nguyen (2019) found evidence for a revived working memory in older subjects when only left PFC and temporal cortex have been synchronized.

Because these studies all refer to working memory, one could argue that the lack of effect in our study is caused by using this montage for the cognitive domain of declarative memory. Targeting HPC and PFC in phase may have suppressed cross-hemispheric processing because of the 180° phase difference between hemispheres. However, studies have shown that the theta frequency shows a clear lateralization (Osipova et al., 2006; Summerfield & Mangels, 2005). Furthermore, two research groups (Burke et al., 2013; Solomon et al., 2019) showed that theta in particular is relevant for establishing synchronization between the areas within a hemisphere, whereas Alekseichuk & colleagues (2017) could show that interhemispheric communication does not seem to play a role, at least for working memory. Moreover, they were able to show that the desynchronization of the two hemispheres tends to lead to a deterioration in performance.

Our study extends the findings with working memory by showing that in-phase theta-tACS can impact long-term memory performance positively when encoding takes places *after* the electrical stimulation. In contrast, in-phase stimulation *during* encoding or retrieval did not benefit long-term memory formation in our study.

The reason that we specifically observed memory benefits of theta-tACS only after, but not during, the stimulation is unclear. Studies using in-phase stimulation in working memory observed their effects during ongoing stimulations (online protocol). The same is applicable to studies on declarative memory, which was the reason why we also decided to use an online protocol (Klink et al., 2020a, 2020b) Even if tACS also alters the membrane potential of neurons toward depolarization and polarization like transcranial direct current stimulation (tDCS), the current is not strong enough to change the rate of action potentials, such as tDCS (Krause et al., 2019). Thus, it rather controls their timing in a frequency- and location-specific manner, which is the reason that tACS is widely used to prove the functional relevance of a certain frequency band in a specific task. This makes tACS, unlike tDCS, particularly relevant in online settings, as it specifically affects intrinsic brain activity.

However, aftereffects of tACS in cognitive performance have been commonly examined as well: in intelligence (Neubauer et al., 2017); in auditory perception (Moliadze et al., 2019); in procedural (Fresnoza et al., 2020; Harada et al., 2020; Sugata et al., 2018); and working memory (Hu et al., 2022; Jaušovec et al., 2014). Interestingly, Kasten and Herrmann (2017) have shown that performance in a mental rotation task can be positively influenced by tACS and the behavioral effect outlasted the end of stimulation, lasting up to 50 min after stimulation. In another study, Fresnoza et al. (2020) showed effects that lasted up to 120 min. Although many studies addressing different cognitive domains have shown a benefit in performance with an in-phase tACS/online protocol, we were the first who found a performance advantage only in learning after the application of theta-tACS (offline protocol), but not with tACS during learning.

So far, the majority of tACS-studies that observed an improvement of declarative memory performance have used an antiphase protocol. In particular, studies with young adults targeting the right hemisphere only (specifically, parietal areas) during encoding showed a tACS-based advantage in memory performance (Alekseichuk et al., 2019a, 2019b; Lang et al., 2019). The left hemisphere as a target shows indifferent results with no benefit in young adults (Antonenko et al., 2016; Meng et al., 2021) but with older subjects (Antonenko et al., 2016; Klink et al., 2020a, 2020b)—even when the midline was targeted (Varastegan et al., 2022). So far, Marko & colleagues have been the only group showing a benefit in performance with an online/in-phase protocol, but for delayed recall only (Marko et al., 2019).

Despite the different stimulation settings, some stimulation parameters show similarities to our study. For example, there is the use of lower theta (4–6 Hz; similar to ours: 5.1–5.3 Hz on average), which tends to improve accuracy during encoding (Alekseichuk et al., 2019a, 2019b; Lang et al., 2019; Varastegan et al., 2022), as well as retrieval (Marko et al., 2019) compared with higher theta (Bender et al., 2019). Furthermore, it should be mentioned that Marko et al. (2019) could already show a positive effect at 1.5 mA peak-to-peak, which is more in line with our setting (2 mA peak-to-peak), even if higher current strengths were mostly used, which showed a clear advantage for memory performance (3–4 mA; Alekseichuk et al., 2019a, 2019b; Lang et al., 2019).

Therefore, it appears that despite the many overlaps with protocols of other studies, an in-phase approach combined with an online protocol does not lead to the desired result of improving declarative memory performance, at least not during stimulation. It seems that such a setting only has a positive effect on working memory, because of the synchronization of the two brain areas addressed by tACS, which has been demonstrated to a certain extent (Alekseichuk et al., 2017; Violante et al., 2017). Since we were not able to find any evidence for an influence of tACS on brain activity, the question remains whether the setting we chose is useful for the question of communication between PFC and HPC in the formation of declarative memory traces.

One of the reasons that we did not observe the expected effects can be that our choice of stimulation setup and our goal to increase PFC-hippocampus coherence was not ideal to improve general memory performance. In contrast to the studies using anti-phase protocols, the specific nature of the in-phase tACS used in our study to synchronize PFC and hippocampus could have caused an irritation in the communication between both brain areas. Based on the source-receiver approach (Sirota et al., 2008) where only the recipient structure is in charge to establish the connection between both brain areas, it could be not necessary to synchronize PFC and hippocampus via in-phase tACS to improve memory performance. Conversely, the flow of information from the source to the receiver itself also takes time, which was not reflected in our setting of a 0° synchronization in each hemisphere. A phase shift of the electrode over the receiver region compared with the electrode over the source could take this into account and might lead to an improvement of the memory performance by generating a travelling wave from the source to the receiver (Alekseichuk et al., 2019a, 2019b). A third explanation could be the use of the beta band as control stimulation, because there is evidence that the beta band has a similarly important role in memory formation processes as the theta band (Brincat & Miller, 2015; Das & Mignon 2021). Thus, we cannot exclude that the lack of difference in memory performance between theta-tACS and control (at least in online stimulation) was due to the role of both frequency bands in declarative memory formation processes. However, the fact that we found an effect after the stimulation had already been administered probably has to do with a special feature of the theta frequency band.

Theta-tACS might have triggered effects such as long-term potentiation (LTP), which lead to the different memory performance during and after tACS (Vossen et al., 2015), especially because LTP is mainly triggered by theta (Wolfgang Klimesch, 1999). Even though we did not find effects at the neurophysiological level, it might be that theta-tACS had an effect at a deeper neuronal level (e.g., neuronal connections). Any improvement in memory performance therefore could only occur after the application of tACS, when the neuronal connections have been strengthened at a sufficient level, which took time. However, the positive outcome in the second list as a consequence from the assumed LTP-like effects is nonetheless a highly relevant result. Because electrical stimulation can only have an effect where the neuronal generators necessary for the corresponding frequency band exist (for the theta band, these are located in the PFC and hippocampus), and bsince we know that communication during learning originates in the hippocampus (Sirota et al., 2008; Das & Menon, 2021), we assume that we may have reached the hippocampus, at least to a certain extent. There were positive, theta-based effects during learning of the second list, which in our eyes speaks for an existing, LTP-like influence of the alternating current on the hippocampus, even if we were not able to provide the neurophysiological evidence for it.

Our results show that, similar to the alpha band (Kasten et al., 2016; Vossen et al., 2015), long-lasting effects may be possible with theta band as well. Especially with regard to theta's role in memory formation, this is a finding that could play a larger role in the future, because longer-lasting effects that outlast the duration of the intervention are highly appreciated for the development of clinically relevant settings, e.g., for the treatment of memory disorders.

### Limitations

Even though this study was planned very carefully, during the data collection we faced unexpected problems due to the SARS-CoV-2 pandemic. Therefore, we did not reach the calculated numbers of participants of 36 in each group (*encoding group*: 30; *retrieval group:* 23), which might explain that some results were only close to significance or that we found no strong effects.

Furthermore, we are only able to argue on the existing literature what might have caused the offline effect, because no data during stimulation were available, we have no proof of any changes on the neurophysiological level to underpin our findings. Although similar to ours, many studies have not found any improvements or any effects at all on brain activity after the use of theta-tACS (Hsu et al., 2017, 2019; Kleinert et al., 2017; Wischnewski et al., 2016). The reason could be that in these studies the goal was to demonstrate the functional relevance of task-related theta by tACS—but because of stimulation artifacts, one could only examine theta during rest after the task. The influence of tACS on brain activity, conversely, is dependent on specific brain states (Kasten & Herrmann, 2022), which might make it difficult to detect a change in task-based theta by tACS beyond electrical stimulation. Thus, the lack of such findings will always raise questions about whether tACS is the actual reason for the cognitive effect found, especially if one argues that the change in brain activity is the reason, which has been found so often for alpha-tACS but very indifferently for other frequency bands.

It is well known that being in-phase with intrinsic brain activity plays an important role (Polanía et al., 2012; Reinhart, 2017; Violante et al., 2017). Similar to these studies, our permanent stimulation should ensure that the intrinsic brain activity adapts to the input from tACS, which is more likely the closer the stimulation frequency is to that of the intrinsic activity (Herrmann & Strüber, 2017). Even though our study showed a benefit after the application of in-phase tACS, we did not take into account the temporal delay between brain areas caused by the migration of specific brain activities (Zhang et al., 2018), which might play a large role in the communication between prefrontal and tempoparietal areas.

In addition, we are not able to verify whether the alternating current reached the hippocampus. When planning the study, we used the Matlab-based roast toolbox to calculate the best fitting electrode positions for our purpose. However, given the individual differences in brain structure, this will only be an approximation of the actual electric field strengths and topologies (Kasten et al., 2019). Furthermore, it might be that even with well-trained experimenters, we may not have been accurate enough to reach the targeted areas (Caulfield et al., 2022), making neuro-navigated electrode placement essential in future studies. Another approach could be the temporal interference stimulation technique, which is able to reach more precisely deeper brain regions, without affecting superficial, off-target structures (Grossman et al., 2017).

Another limitation of the study is the lack of a real sham stimulation. Especially with regard to the role of both frequency bands in memory formation processes, it would be helpful for the interpretation of the results if there were performance scores without any influence of stimulation. Because of our design, it is possible to use the values from the immediate recall of the retrieval group as sham values for the immediate recall of the encoding group for a comparison. However, this possibility is completely missing for the *retrieval group*. The performance in the delayed recall of the *encoding group* must always be considered in context of the existing influence of stimulation during learning.

## Conclusions

Successful learning or recall of information requires, among other things, effective communication between the prefrontal cortex and the hippocampus, and our study was intended to show that the theta band activity plays a key role in this. Even if we failed to show neurophysiological support, our results revealed that the functional proof of this link is frequency-specific and highly dependent on the use of online or offline approaches. Because we only found this effect for word pairs learned after using theta-tACS, targeting PFC and the hippocampus simultaneously with tACS does not seem to be optimal when it comes to declarative memory. Especially when most of the studies reporting beneficial effects only targeted one brain area at the time. We argue that, in conjunction with other studies, our results support the assumption that the time window, which is critical for successful transmission of information, is initiated from the respective receiving structure (either PFC or the hippocampus). This also might explain why some assume that an encoding-related increase of theta is only a reflection of activity in the hippocampus (Kahana et al., 2001). Therefore, future studies should systematically investigate the role of the source and recipient of information in the communication between PFC and the hippocampus and to what extent this depends on the hemisphere. In addition, coherence analyses would provide deeper insights into possible effects caused by tACS at the network level.

More than this, the role of theta in the formation of declarative memory traces should not be considered in isolation from its link to the gamma frequency band, and future studies should take this into account as well. The multiplex buffer model described, among other things, that the stored elements are organized by theta-nested gamma subcycles (Lisman & Jensen, 2013), which was demonstrated in a study by Alekseichuk & colleagues (2016) but for working memory only. Thus, their cross-frequency tACS approach (bursts of high gamma oscillations nested in theta waves) could be also relevant to study the formation of declarative memory traces. Nevertheless, our unexpected effects open up opportunities to consider theta-tACS as a possible approach in the development of treatment approaches for memory disorders, which also needs further exploration.

## Supplementary Information

Below is the link to the electronic supplementary material.

Supplementary file1 (DOCX 4784 kb)

## Declarations

### Data Availability

Not applicable (see section above).
