# Supplementary material for: Differential online and offline effects of theta-tACS on memory encoding and retrieval
Source: Cogn Affect Behav Neurosci. 2024 Jul 31;24(5):894–911. doi: 10.3758/s13415-024-01204-w (PMC11390785; doi:10.3758/s13415-024-01204-w)
Supplement: Supplementary file 1 — Supplementary file1 (DOCX 4784 kb) [file 13415_2024_1204_MOESM1_ESM.docx]

Differential on- and offline effects of theta-tACS on memory encoding and retrieval

For submission to Cognitive, Affective and Behavioral Neuroscience

Sven Paßmann^1,2^, Sandrine Baselgia^1^, Florian H. Kasten^4,5^, Christoph S. Herrmann^3^, Björn Rasch^1^

^1^ Cognitive Biopsychology and Methods, Department of Psychology, Université Fribourg, Fribourg, Switzerland

^2^ Department of Neurology, University Medicine Greifswald, Greifswald, Germany (present adress)

^3^ Experimental Psychology Lab, Department of Psychology, Carl von Ossietzky Universität, Oldenburg, Germany

4 Centre de Recherche Cerveau & Cognition, CNRS, Toulouse, France

5 Université Toulouse III Paul Sabatier, Toulouse, France

Corresponding Author:

Sven Paßmann, University of Fribourg, Department of Biopsychology and Methods, Rue P.-A.-de-Faucigny 2, 1700 Fribourg, Switzerland

e-mail: sven.passmann@unifr.ch

or

Björn Rasch, University of Fribourg, Department of Biopsychology and Methods, Rue P.-A.-de-Faucigny 2, 1700 Fribourg, Switzerland

Tel. +41 26 300 7637, e-mail: bjoern.rasch@unifr.ch

**Supplemental information**

**Supplemental Table S1**

*Current distribution at the target areas*

|  | Prefrontal cortex | | | | | | | | Hippocampal area | | | | | | |  |
| --- | --- | --- | --- | --- | --- | --- | --- | --- | --- | --- | --- | --- | --- | --- | --- | --- |
|  | left | | | right | | | | | left | | | right | | | |  |
| Target + Return | max | mean | | | max | | mean | | max | | mean | max | | | mean |  |
| FP2/P8 + CP5 | 0.116 | | 0.093 | | | 0.099 | | 0.074 | 0.139 | 0.110 | | | 0.091 | 0.091 | | |
| FP2/TP8 + TP7 | 0.110 | | 0.087 | | | 0.102 | | 0.075 | 0.144 | 0.125 | | | 0.118 | 0.118 | | |
| FP2/P8 + P7 | 0.083 | | 0.067 | | | 0.083 | | 0.062 | 0.113 | 0.096 | | | 0.098 | 0.098 | | |
| FP2/CP6 + P7 | 0.092 | | 0.072 | | | 0.092 | | 0.066 | 0.114 | 0.101 | | | 0.118 | 0.118 | | |
| F4/TP8 + CP5 | 0.143 | | 0.109 | | | 0.149 | | 0.108 | 0.148 | 0.117 | | | 0.120 | 0.120 | | |
| Afz/TP8 + Pz | 0.120 | | 0.096 | | | 0.112 | | 0.084 | 0.096 | 0.069 | | | 0.097 | 0.097 | | |
| FP1/TP7 + FP2/TP8 | 0.168 | | 0.124 | | | 0.175 | | 0.127 | 0.197 | 0.168 | | | 0.194 | 0.194 | | |
| FP1/P7 + FP2/P8 | 0.215 | | 0.154 | | | 0.207 | | 0.148 | 0.147 | 0.123 | | | 0.175 | 0.175 | | |

*Note.* Simulation of the current distribution, according to the stimulation electrode arrangement and sorted by regions of interest (8 out of 12 simulated electrode settings). Please note that due to broken original data the simulations had to be performed again with the package simNIBS (version 4.0) using Python Version 3.9 and the human connectome atlas (Glasser et al., 2016). Even though better results could have been achieved in the hippocampal area with the electrode setup FP1/TP7 + FP2/TP8, we decided - due to the electrode size of 5x5 cm and the associated poorer attachment of this setup - in favor of the subsequent setup (FP1/P7 + FP2/P8), which showed approximately similar results. Results are shown as V/m.

**Supplemental Table S2**

*Correlations between immediate and delayed recall*

|  | Encoding group (N = 30) | | | | | | | Retrieval group (N = 23) | | | | | |  |
| --- | --- | --- | --- | --- | --- | --- | --- | --- | --- | --- | --- | --- | --- | --- |
|  | theta-tACS | | | beta- tACS | | | theta-tACS | | | | beta- tACS | | | |
|  | DoF | *r* | ***p*** | DoF | *r* | ***p*** | DoF | | *r* | ***p*** | DoF | *r* | ***p*** | |
| General analyses | 28 | 16.72 | **<.001** | 28 | 17.17 | **<.001** | 21 | | 22.31 | **<.001** | 21 | 12.65 | **<.001** | |
|  |  |  |  |  |  |  |  | |  |  |  |  |  | |
| Explorative analyses |  |  |  |  |  |  |  | |  |  |  |  |  | |
| List 1 | 28 | 12.43 | **<.001** | 28 | 17.06 | **<.001** | 21 | | 14.79 | **<.001** | 21 | 7.74 | **<.001** | |
| List 2 | 28 | 19.02 | **<.001** | 28 | 14.88 | **<.001** | 21 | | 12.93 | **<.001** | 21 | 13.83 | **<.001** | |

*Note.* Pearson’s correlation calculated separately for general (section 3.1) and exploratory analyses (section 3.2). Calculations have been executed between immediate and delayed recall, separately for stimulation conditions, groups and lists. The correlation analyses show that the individual performance of the test subjects does not exhibit any extreme changes between immediate and delayed recall. DoF, degrees of freedom; *r*, r statistic; *p*, p-Value.

**Supplemental Table S3**

*Bayesian rmANOVA with factors stimulation condition, time, and group*

|  | | | | | | | |
| --- | --- | --- | --- | --- | --- | --- | --- |
| **Effects** | | **P(incl)** | | **P(incl\|data)** | | **BF_Inclusion_** | |
| StimCon |  | 0.2632 |  | 0.1199 |  | 0.145 |  |
| Time |  | 0.2632 |  | 0.7869 |  | 10.048 |  |
| Group |  | 0.2632 |  | 0.2963 |  | 0.507 |  |
| StimCon ✻ Time |  | 0.2632 |  | 0.0358 |  | 0.287 |  |
| StimCon ✻ Group |  | 0.2632 |  | 0.0207 |  | 0.335 |  |
| Time ✻ Group |  | 0.2632 |  | 0.1036 |  | 0.363 |  |
| StimCon ✻ Time ✻ Group |  | 0.0526 |  | 3.05e-4 |  | 0.264 |  |
| *Note*. Analysis of effects, obtained from Bayesian repeated measures ANOVA. Compares models that contain the effect to equivalent models stripped of the effect. Higher-order interactions are excluded. Analysis suggested by Sebastiaan Mathôt. BF, Bayes factor. | | | | | | | |

**Supplemental Table S4**

*Bayesian rmANOVA with factors time and group for theta-tACS*

| **Effects** | | **P(incl)** | | **P(incl\|data)** | | **BF_Inclusion_** | |
| --- | --- | --- | --- | --- | --- | --- | --- |
| Time |  | 0.400 |  | 0.702 |  | 13348.545 |  |
| Group |  | 0.400 |  | 0.291 |  | 0.708 |  |
| Time ✻ Group |  | 0.200 |  | 0.298 |  | 1.024 |  |
| *Note.* Analysis of effects, obtained from Bayesian repeated measures ANOVA. Compares models that contain the effect to equivalent models stripped of the effect. Higher-order interactions are excluded. Analysis suggested by Sebastiaan Mathôt. BF, Bayes factor. | | | | | | | |

**Supplemental Table S5**

*Bayesian rmANOVA with factors time and group for beta-tACS*

| **Effects** | | **P(incl)** | | **P(incl\|data)** | | **BF_Inclusion_** | |
| --- | --- | --- | --- | --- | --- | --- | --- |
| Time |  | 0.400 |  | 0.687 |  | 7.993 |  |
| Group |  | 0.400 |  | 0.301 |  | 0.637 |  |
| Time ✻ Group |  | 0.200 |  | 0.227 |  | 0.849 |  |
| \| *Note.* Analysis of effects, obtained from Bayesian repeated measures ANOVA. Compares models that contain the effect to equivalent models stripped of the effect. Higher-order interactions are excluded. Analysis suggested by Sebastiaan Mathôt. BF, Bayes factor. \| \| --- \| | | | | | | | |

**Supplemental Table S6**

*Bayesian rmANOVA with factors stimulation condition, time, list and group*

|  | | | | | | | |
| --- | --- | --- | --- | --- | --- | --- | --- |
| **Effects** | | **P(incl)** | | **P(incl\|data)** | | **BF_Inclusion_** | |
| Stim |  | 0.11377 |  | 0.08725 |  | 0.110 |  |
| Time |  | 0.11377 |  | 0.58625 |  | 3.975 |  |
| List |  | 0.11377 |  | 0.45009 |  | 11.300 |  |
| Group |  | 0.11377 |  | 0.14858 |  | 0.366 |  |
| Stim ✻ Time |  | 0.29940 |  | 0.03234 |  | 0.216 |  |
| Stim ✻ List |  | 0.29940 |  | 0.02062 |  | 0.188 |  |
| Time ✻ List |  | 0.29940 |  | 0.15781 |  | 0.239 |  |
| Stim ✻ Group |  | 0.29940 |  | 0.01497 |  | 0.224 |  |
| Time ✻ Group |  | 0.29940 |  | 0.10233 |  | 0.249 |  |
| List ✻ Group |  | 0.29940 |  | 0.33427 |  | 1.944 |  |
| Stim ✻ Time ✻ List |  | 0.11377 |  | 6.23e-4 |  | 0.177 |  |
| Stim ✻ Time ✻ Group |  | 0.11377 |  | 6.45e-4 |  | 0.203 |  |
| Stim ✻ List ✻ Group |  | 0.11377 |  | 0.07340 |  | 48.304 |  |
| Time ✻ List ✻ Group |  | 0.11377 |  | 0.00269 |  | 0.197 |  |
| Stim ✻ Time ✻ List ✻ Group |  | 0.00599 |  | 1.21e-6 |  | 0.298 |  |
| *Notes*. Analysis of effects, obtained from Bayesian repeated measures ANOVA. Compares models that contain the effect to equivalent models stripped of the effect. Higher-order interactions are excluded. Analysis suggested by Sebastiaan Mathôt. Results showed very strong evidence (BF_incl_) in favor of a GROUP x STIM x LIST interaction and strong evidence for the main factor LIST. No evidence was found for the LIST x TIME interaction. BF, Bayes factor. | | | | | | | |
|  | | | | | | | |

**Supplemental Table S7**

*Bayesian rmANOVA with factors stimulation condition, time and group for list 1*

|  | | | | | | | |
| --- | --- | --- | --- | --- | --- | --- | --- |
| **Effects** | | **P(incl)** | | **P(incl\|data)** | | **BF_Inclusion_** | |
| Stim |  | 0.2632 |  | 0.1257 |  | 0.155 |  |
| Time |  | 0.2632 |  | 0.7178 |  | 3.750 |  |
| Group |  | 0.2632 |  | 0.2363 |  | 0.352 |  |
| Stim ✻ Time |  | 0.2632 |  | 0.0308 |  | 0.243 |  |
| Stim ✻ Group |  | 0.2632 |  | 0.0372 |  | 0.809 |  |
| Time ✻ Group |  | 0.2632 |  | 0.0629 |  | 0.297 |  |
| Stim ✻ Time ✻ Group |  | 0.0526 |  | 4.47e-4 |  | 0.282 |  |
| *Notes*. Analysis of effects, obtained from Bayesian repeated measures ANOVA. Compares models that contain the effect to equivalent models stripped of the effect. Higher-order interactions are excluded. Analysis suggested by Sebastiaan Mathôt. Results showed no evidence for any main factor or interaction. BF, Bayes factor. | | | | | | | |
|  | | | | | | | |

**Supplemental Table S8**

*Bayesian rmANOVA with factors stimulation condition, time and group for list 2*

|  | | | | | | | |
| --- | --- | --- | --- | --- | --- | --- | --- |
| **Effects** | | **P(incl)** | | **P(incl\|data)** | | **BF_Inclusion_** | |
| Stim |  | 0.2632 |  | 0.02022 |  | 0.197 |  |
| Time |  | 0.2632 |  | 0.24969 |  | 0.402 |  |
| Group |  | 0.2632 |  | 0.04233 |  | 0.535 |  |
| Stim ✻ Time |  | 0.2632 |  | 0.06587 |  | 0.237 |  |
| Stim ✻ Group |  | 0.2632 |  | 0.87181 |  | 110.121 |  |
| Time ✻ Group |  | 0.2632 |  | 0.07296 |  | 0.262 |  |
| Stim ✻ Time ✻ Group |  | 0.0526 |  | 0.00382 |  | 0.281 |  |
| *Notes*. Analysis of effects, obtained from Bayesian repeated measures ANOVA. Compares models that contain the effect to equivalent models stripped of the effect. Higher-order interactions are excluded. Analysis suggested by Sebastiaan Mathôt. Results showed extreme evidence (BF_incl_) in favor of a GROUP x STIM interaction. BF, Bayes factor. | | | | | | | |
|  | | | | | | | |

**Supplemental Table S9**

*Mood and sleep habit questionnaires, based on session*

|  | Encoding group (N = 30) | | | | | Retrieval group (N = 23) | | | | |
| --- | --- | --- | --- | --- | --- | --- | --- | --- | --- | --- |
|  | Session 1 | | Session 2 | | *p* | Session 1 | | Session 2 | | *p* |
| SF-A/R | *M* | *SD* | *M* | *SD* |  | *M* | *SD* | *M* | *SD* |  |
| ESS | 3 | 1.7 | 3.2 | 1.2 | .36 ^+^ | 3 | 1 | 2.5 | .9 | .09 ^+^ |
| DSS | 1.6 | .6 | 1.5 | .7 | .37 ^+^ | 1.9 | 1.1 | 1.5 | .7 | .34 ^t^ |
| VZA | 1.4 | 1.2 | 1.7 | 1.5 | .42 ^t^ | 1.4 | 1.2 | 1.4 | 1.2 | .1 ^t^ |
| ASC | 7.8 | 3.4 | 4 | .7 | .34 ^+^ | 4 | .9 | 4.2 | .7 | .91 ^+^ |
| GSD | 7.4 | 1.9 | 6. | 2.4 | .07 ^t^ | 7.5 | 1.3 | 7.4 | 1.2 | .64 ^+^ |
| SQ | 12.3 | 6.4 | 3.9 | .6 | .26 ^+^ | 4 | .7 | 4.2 | .5 | .14 ^+^ |
| GES | 9.1 | 4.8 | 3.3 | .6 | .12 ^+^ | 3.3 | .8 | 3.4 | .8 | .56 ^t^ |
| PSYA | 8.3 | 3.4 | 3. | .8 | .57 ^+^ | 3.7 | .7 | 3.8 | .6 | .67 ^+^ |
| PSYE | 7.5 | 4.3 | 3.2 | .9 | .35 ^+^ | 3 | .6 | 3.2 | .8 | .3 ^+^ |
| PSS | 1.4 | .5 | 1.4 | .4 | .69 ^t^ | 1.3 | .5 | 1.2 | .3 | .48 ^t^ |
|  |  | |  | |  |  | |  | |  |
| MDBF |  | |  | |  |  | |  | |  |
| GS | 17.8 | 2.2 | 17. | 1.8 | .2 ^+^ | 17. | 2.2 | 17.3 | 1.8 | .58 ^t^ |
| WM | 14.3 | 3.3 | 13. | 2.2 | .79 ^t^ | 14.3 | 1.9 | 13.8 | 3.4 | .48 ^+^ |
| RU | 17. | 2 | 16. | 2.6 | .04 ^+^ | 16.5 | 2.7 | 17 | 2 | .21 ^+^ |

*Note*. All values are depicted as mean values ± standard deviations. Significant differences are highlighted in bold. *SF-A/R:* ASC, general sleep characteristic; DSS, difficulty sleeping through; ESS, difficulty falling asleep; GES, feeling of recovery; GSD, total sleep time; PSS, psychosomatic symptoms during the sleep phase, PSYA, mental balance before going to sleep; PSYE, being mentally exhausted before going to bed; SQ, sleep quality; VZA, premature awakening. *MDBF*: GS, good/bad mood; WM, awake/tired; RU, rest/unrest mood. *p*, p-Value. ^t^ Wilcoxon rank test. ^+^paired associate test.

**Supplemental Table S10**

*Mood and sleep habit questionnaires, based on stimulation condition*

|  | Encoding group (N = 30) | | | | | Retrieval group (N = 23) | | | | |
| --- | --- | --- | --- | --- | --- | --- | --- | --- | --- | --- |
|  | theta-tACS | | beta- tACS | | *p* | theta-tACS | | beta- tACS | | *p* |
| SF-A/R | *M* | *SD* | *M* | *SD* |  | *M* | *SD* | *M* | *SD* |  |
| ESS | 3 | 1.2 | 3.2 | 1.2 | .363^+^ | 2.7 | .8 | 2. | 1.1 | .589^t^ |
| DSS | 1.7 | .7 | 1.5 | .6 | .155^+^ | 1.5 | .6 | 1.8 | 1.1 | .569^t^ |
| VZA | 1.4 | 1.2 | 1.7 | 1.5 | .424^t^ | 1.4 | 1.2 | 1.4 | 1.2 | 1.0^t^ |
| ASC | 4.1 | .6 | 3. | .7 | .196^+^ | 4. | .8 | 4 | .9 | .466^t^ |
| GSD | 6.9 | 2.4 | 7.2 | 2 | .905^t^ | 7.2 | 1.3 | 7.7 | 1 | .099^t^ |
| SQ | 4 | .4 | 3.9 | .6 | .256^+^ | 4.2 | .4 | 4 | .8 | .559^t^ |
| GES | 3.4 | .6 | 3.4 | .8 | .464^+^ | 3.3 | .8 | 3.4 | .7 | .403^+^ |
| PSYA | 3.6 | .8 | 3.5 | .8 | .563^+^ | 3.8 | .6 | 3.7 | .7 | .585^+^ |
| PSYE | 3.1 | .8 | 3.1 | .8 | .883^+^ | 3.2 | .7 | 3 | .7 | .243^+^ |
| PSS | 1.4 | .5 | 1.4 | .4 | .720^t^ | 1.3 | .3 | 1.3 | .3 | .673^t^ |
|  |  | |  | |  |  | |  | |  |
| MDBF |  | |  | |  |  | |  | |  |
| GS | 17.8 | 2.2 | 17.8 | 1.8 | .913^t^ | 18.1 | 1.7 | 16.7 | 2.2 | .001^+^ |
| WM | 14.2 | 2.2 | 13.8 | 3.3 | .509^+^ | 13.8 | 2.9 | 14.3 | 2.5 | .479^+^ |
| RU | 16.7 | 2.4 | 16.8 | 2.3 | .787^+^ | 17.1 | 2.1 | 16.4 | 2.7 | .097^+^ |

*Note*. All values are depicted as mean values ± standard deviations. Significant differences are highlighted in bold. *SF-A/R:* ASC, general sleep characteristic; DSS, difficulty sleeping through; ESS, difficulty falling asleep; GES, feeling of recovery; GSD, total sleep time; PSS, psychosomatic symptoms during the sleep phase, PSYA, mental balance before going to sleep; PSYE, being mentally exhausted before going to bed; SQ, sleep quality; VZA, premature awakening. *MDBF*: GS, good/bad mood; WM, awake/tired; RU, rest/unrest mood. *p*, p-Value. ^t^ Wilcoxon rank test. ^+^paired associate test.

**Supplemental Table S11**

*Adverse effects – sensation ratings*

|  | Encoding group (N = 30) | | | | | Retrieval group (N = 23) | | | | |
| --- | --- | --- | --- | --- | --- | --- | --- | --- | --- | --- |
|  | theta-tACS | | beta-tACS | | *p* | theta-tACS | | beta-tACS | | *p* |
| Adverse effects | *M* | *SD* | *M* | *SD* |  | *M* | *SD* | *M* | *SD* |  |
| Phosphenes | 0.7 | 1.2 | 1.8 | 1.6 | .002 ^+^ | 2 | 1 | 3. | .8 | .18 ^+^ |
| Pain | 0.8 | 1.2 | 0.9 | 1.3 | .84 ^t^ | 1.8 | 1.2 | 1.6 | 1 | 1.00 ^t^ |
| Tingling | 1.7 | 1.4 | 1.9 | 1.3 | .66 ^+^ | 1.6 | .7 | 2.2 | 1.1 | NA ^+^ |
| Tickling | 0.6 | 1 | 0.9 | 1.1 | .17 ^t^ | 1.5 | 1.6 | 2.3 | 1 | .23 ^+^ |
| Burn | 0.6 | 1 | 0.7 | 1.2 | .78 ^t^ | 1.6 | 1.1 | 2.3 | .6 | .17 ^t^ |
| Tired | 1.3 | 1.3 | 1.5 | 1.5 | .53 ^+^ | 1.9 | .7 | 1.9 | .8 | .52 ^+^ |
| Nervousness | 0.3 | .8 | 0.6 | 1.2 | .40 ^t^ | 2.2 | 1.5 | 2.3 | 1.4 | .34 ^+^ |
| Concentration | 1.2 | 1.7 | 1. | 1.4 | .16 ^t^ | 1. | .8 | 2.1 | 1.4 | .62 ^+^ |
| Visual Sensations | 0.1 | .4 | 0.9 | 1.3 | .006 ^t^ | 1.3 | .6 | 1.8 | 1 | 1.00 ^t^ |
| Headaches | 0.5 | 1 | 0.6 | 1.1 | .62 ^t^ | 1.3 | .7 | 2 | 1.2 | .5 ^+^ |

*Note.* All values are depicted as mean values ± standard deviations. Significant differences are highlighted in bold. *p*, p-Value. *NA*, not applicable due to insufficient numbers of ratings. ^t^ Wilcoxon rank test. ^+^paired associate test.

Additional behavioral analyses and discussion

In addition to the main analyses, we conducted a 3-factorial repeated-measures analysis of variances with the within-subject factors GROUP (*encoding/retrieval*) STIM (theta‑/beta‑tACS) and LIST (online/offline recall) on two different measures, *Gains* and *Losses*. Based on the *Hits* from the main analysis, we considered items as *Gains* when they have not been retrieved during immediate, but have been retrieved successfully during delayed recall. And we considered items as *Losses*, when they have been retrieved successfully during immediate, but not delayed recall. Similar to the main analysis, we conducted exploratory analyses for both groups by extending this approach for lists (factor LIST: online/offline) while contrasting the *encoding* against the *retrieval group*.

The 3-factorial rmANOVA for *Gains* revealed significance only for the factor STIM (*F_(1,51)_= 4.95, p = .033, η2 = .04,* see figure **S1**), showing that in theta-tACS, participants in both groups had less *Gains* than under control stimulation. This effect disappeared when analyzed separately for each group (all p > .124). For *Losses,* the results showed a significant effect in GROUP only (*F_(1,51)_= 4.01, p = .049, η2 = .05,* see figure **S1**), indicating a larger likelihood to forget items in the *retrieval* compared to the *encoding group* no matter the stimulation condition (all remaining p > .732).

The exploratory analyses for *Gains* including the factor LIST revealed again significance in the factor STIM (*F_(1,51)_= 4.95, p = .031, η2 = .03,* see figure **S2**), showing less *Gains* under theta-tACS compared to control stimulation. In addition, the factor LIST showed a significance as well (*F_(1,51)_9.6, p = .003, η2 = .05*), indicating that items from list 2 (items have been learned after the participant received the stimulation) were more likely to be remembered at a later time point no matter the stimulation condition. The analysis for each group separately showed that participants in the *encoding group* had more *Gains* in list 2 compared to list 1 (factor LIST: *F_(1,51)_= 5.44, p = .027, η2 = .03*), while in the *retrieval* group this difference was not significant (factor LIST: *F_(1,51)_= 4.13, p = .054, η2 = .03*, all remaining p > .124). The listwise analyses though revealed no effect at all (all remaining p > .107), besides an interesting trend in factor STIM for list 1 (*F_(1,51)_= 3.54, p = .066, η2 = .03*) indicating that under theta-tACS and in both groups participants were more likely to forget the items of list 1 at a later time point that they could not remember during learning.

For *Losses*, our results again showed a significant effect only in the factor GROUP (*F_(1,51)_= 4.08, p = .049, η2 = .05,* see figure **S2***)* indicating that in general participants of the *retrieval group* forgot more items compared to the *encoding group* (all remaining p > .162).

In addition to our main analysis, these results provide an interesting look at a possible mechanism for how items are formed and what frequencies may also play a role in recall. Although it appears that the successful recall of items at a later time point benefited from stimulus-free learning, there is some hint in our data that beta-tACS during learning increased the likelihood that items can be successfully recalled at a later time point, even if they are not remembered during learning. It has already been shown that beta frequency band may play a role in the formation of memory, specifically when participants intended to learn (Schneider & Rose, 2016). Due to our design, it is difficult to interpret the results in terms of improvement or deterioration in each condition and group. The fact that the *Gains* are generally lower under theta-tACS than in the control stimulation could only be clearly assessed as a deterioration (or as a benefit for the control stimulation) in comparison with results without any influence by any stimulation – results which are not available in the present study. In our case, however, the *Gains* in the *encoding group* are influenced either by the stimulation during learning, or in the case of the *retrieval group* by the stimulation during delayed recall.

**Supplemental Figure 1**

*Behavioral results for Gains and Losses*





*Note.* The figure depicts the performance (correct answers in %) in the language learning task of both groups for *Gains* and *Losses*, separately for tACS-conditions (red: theta‑tACS; grey: beta‑tACS). For *Gains*, a tACS-related effect was found without interaction with the factor GROUP. For *Losses*, we found a group related effect only. *Encoding group*, N = 30. *Retrieval group*, N = 23.

**Supplemental Figure 2**

*Behavioral results for Gains and Losses, separated for lists*





*Note.* The figure depicts the performance (correct answers in %) in the language learning task of both groups for *Gains* and *Losses*, separately for tACS-conditions (red: theta‑tACS; grey: beta‑tACS) and lists. In addition to a tACS-related effect, a LIST-related effect was found in *Gains* showing more *Gains* in general for list 2 compared to list 1. For *Losses*, we found again a group related effect only. *Encoding group*, N = 30. *Retrieval group*, N = 23.

Reference supplements

Glasser, M.F., Coalson, T.S., Robinson, E.C., Hacker, C.D., Harwell, J., Yacoub, E., Ugurbil, K., Andersson, J., Beckmann, C.F., Jenkinson, M., et al. (2016). A multi-modal parcellation of human cerebral cortex. Nature 536, 171–178. 10.1038/nature18933.

Schneider, S. L., & Rose, M. (2016). Intention to encode boosts memory-related pre-stimulus EEG beta power. *NeuroImage*, *125*, 978–987. <https://doi.org/10.1016/j.neuroimage.2015.11.024>

[Thielscher, A., Antunes, A. and Saturnino, G.B. (2015), Field modeling for transcranial magnetic stimulation: a useful tool to understand the physiological effects of TMS? IEEE EMBS 2015, Milano, Italy](http://dx.doi.org/10.1109/EMBC.2015.7318340).

Van Rossum, G., & Drake Jr, F. L. (1995). Python reference manual. Centrum voor Wiskunde en Informatica Amsterdam.
